# Partial trisomy 13q22-qter associated to leukoencephalopathy and late onset generalised epilepsy

**DOI:** 10.1186/1755-7682-1-5

**Published:** 2008-04-29

**Authors:** Renee Ribacoba, Manuel Menendez-Gonzalez, Ines Hernando, Javier Salas, Maria Luisa Giros

**Affiliations:** 1 Unit of Neurology, Hospital Alvarez Buylla, Mieres, Spain; 2 Department of Molecular Genetics, Hospital Universitario Central de Asturias, Spain; 3 Department of Neurology, Hospital Universitario Central de Asturias, Oviedo, Spain; 4 Institut of Clinic Biochemistry, Corporació Sanitària Clínic, Barcelona, Spain

## Abstract

The partial trisomy 13q.22 is an uncommon chromosomopathy. We present a case with a partial trisomic component 13q22 and a monosomic component 5p15 from paternal origin. This patient developed early menopause and major neurological disorders as leukoencephalopathy, late onset generalised epilepsy and stroke. She also had fatty acids disturbances and their potential relation to the neurological disorders and early menopause is discussed. The presented case illustrates the phenotype of 13q22-qter in adult age and reaffirms the importance of studying the karyotype of any patient with seizures or leukoencephalopathy particularly when there are associated other clinical features including stroke at a young age, fatty acids disturbances, microcephaly, hypotelorism, short neck, hemangiomata, short fingers or distal swell in thumbs.

## Background

The partial trisomy 13 q is uncommon; its frequency has not been well determined. The few cases described in the medical literature recognised a specific phenotype, however, with extensive variability of expression. Major phenotypic features are: psychomotor retardation, frontal bossing, stubby nose, long philtrum and hemangiomata [1-3]. When the trisomy derives from segment q22 consistent findings are mental retardation, frontal bossing, long upwardly curved eyelashes, ears with small lobules and prominent antehelixes and hemangiomata. Other characteristics such as microcephaly, hypotelorism and hexadactyly, are usual but not constant. [4-8]. In patients with trisomy 13 syndrome holoprosencephaly appears in approximately 80% of cases, but callosal dysgenesis, hippocampal hypoplasia, olfatory hypoplasia, bilateral perisylvian and rolandic cortical dysplasia, vermian hypoplasia, dysplasia of the dentate nucleus have also been reported. The cases that survive often suffer seizures.

We report here a partial trisomy 13q22 case with prominent neurological disorders: leukoencephalopathy, late onset generalised epilepsy and stroke.

## Case presentation

**Figure 1:**
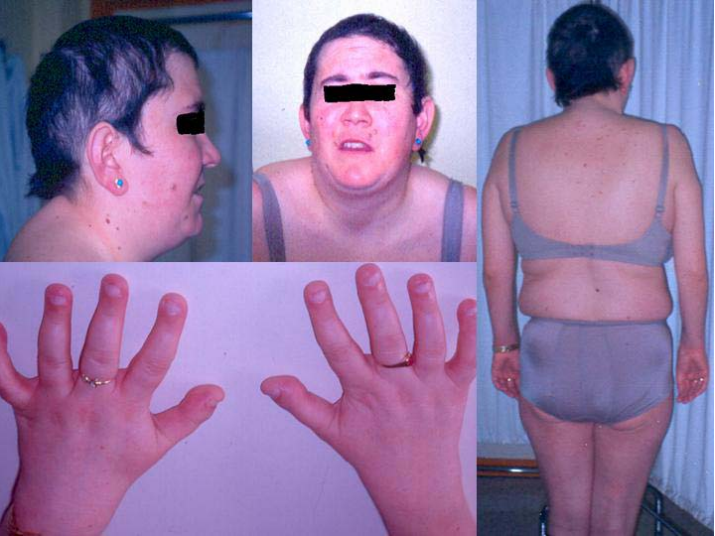
Phenotype of propositus. Head and neck: large ears with prominent antehelix, little hair, hypotelorism and short neck. Skin: acne vulgaris and hemangiomatas. Hands: short fingers and distal deformed in both thumbs. Body: wide shoulders and narrows pelvic girdle.

**Figure 2:**
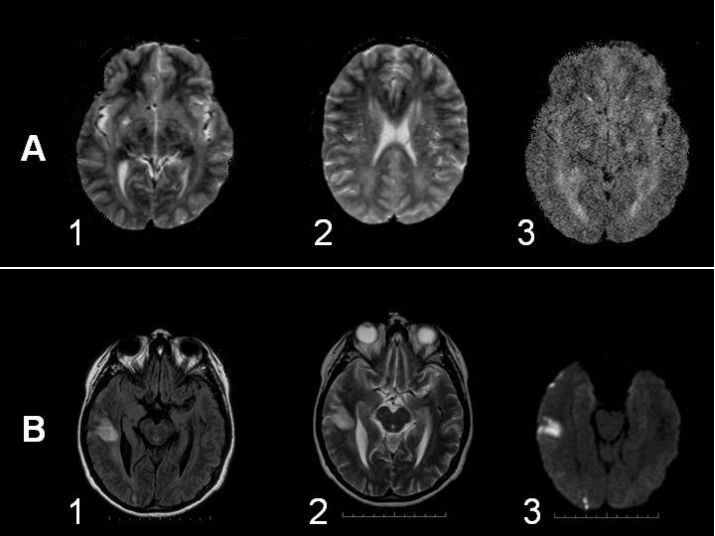
Axial MRI images. A) Basal (pre-stroke): bilateral hyperintense lesions in white matter, predominantly in posterior regions in T2 weighted (1 y 2) and diffusion (3) sequences. B) Post-stroke: hyperintense signal in FLAIR (1), T2 (2) and diffusion (3) sequences localized in the right temporal cortex and subcortical area suggesting an ischemic stroke.

**Figure 3:**
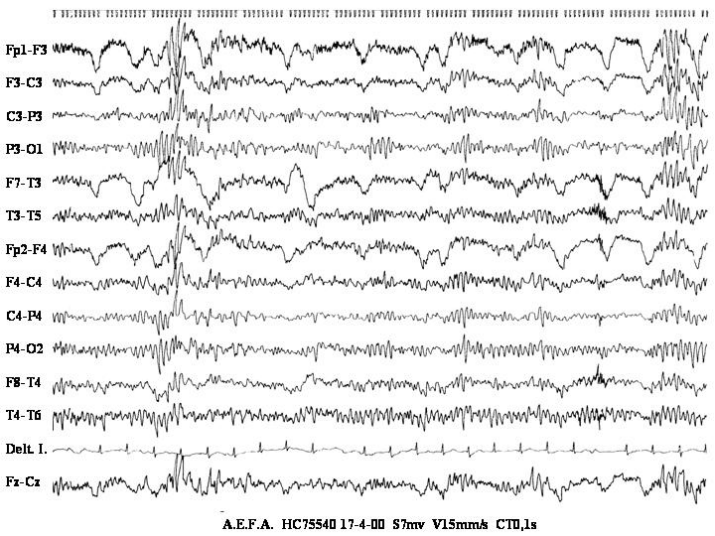
EEG showed normal background activity and polyspike generalised discharges.

We present a 33 years old female, born after a normal pregnancy and delivery of non consanguineous parents. There was no family history of epilepsy. Her father suffers severe peripheral arteriopathy. Motor development was normal. At the age of 13 she dropped out of primary school due to learning disabilities. At age 29 she was menopausal. When she was 30 years old she suffered two isolated unexplained falls; later she suffered a generalised myoclonic seizure that was repeated three times during next eight months and at this point was attended at our hospital. Her phenotype included slight microcephaly and hypotelorism, short neck, small hemangiomata on face and back, android obesity and short fingers with distal swell in both thumbs (fig. 1). She had low IQ and hypoacusia. Abdominal echography showed splenomegaly. Blood tests showed hypochromic anaemia without other relevant abnormalities in haematology or biochemistry including normal renal and liver function. Total cholesterol, LDL-cholesterol and very long chain fatty acids (VLCFA) were also normal in blood. However 26:0/22:0 fatty acids were increased in mononuclear cells while 24:0 and 26:0 fatty acids were normal in fibroblasts. Total cholesterol in lymphocytes was low. Adrenoleukodystrophya-protein was negative in white blood cells. The electrocardiogram, echocardiography, carotid Doppler, conduction speeds and somatosensory evoked potentials were normal. T2 weighted MRI showed bilateral hyperintense lesions in white matter, predominantly in posterior regions (fig. 2A). EEG showed normal background activity with spike/wave and polyspikes generalised discharges that increased during NREM sleep (fig. 3). Visual evoked potential showed low amplitude and abnormal configuration. Brainstem auditive evoked potentials were abnormal with the wave V delayed and the waves I, II and III absent. She was put on valproic acid and EEGs were progressively normalizing.

She suddenly suffered left hemiplegia. MRI (Flair and T2 weighted and diffusion sequences) showed hyperintense signal in the right temporal cortex and subcortical area suggesting an ischemic stroke (fig 2B). Evaluation of the carotid bifurcation with magnetic resonance angiographic techniques showed stenosis (40% calibre) on the beginning of the right internal carotid artery. The hypercoagulability study showed moderate increase of the VIII factor. She was put on clopidogrel 75 mg. per day. During the three next years she only had a nocturnal seizure in relation with oversight of valproic acid intake. Eventually she suffered a nocturnal status epilepticus and died as a result. Unfortunately, the autopsy was not performed.

**Figure 4:**
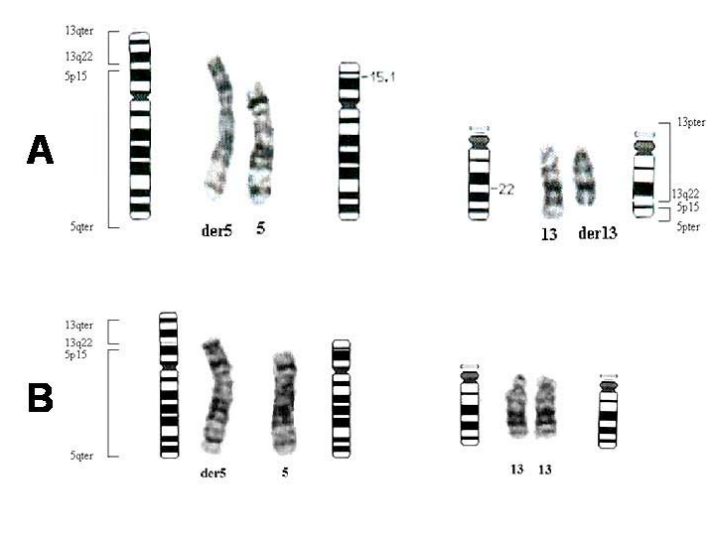
Partial karyotype of the propositus and her father with the corresponding ideogram. A) Propositus's karyotype redesigned as 46,XX,-5,+der5(5qter->5p15::13q22->13qter)t(5;13)pat. There is extra material on short arm of chromosome 5. B). Father's karyotype with translocation (5;13)(p15;q22).

Cytogenic chromosome analyses were performed on G banded metaphases from leukocyte cultures. Excess of chromosomal material on the short arms in one of the chromosomes 5 was detected in the propositus. FISH (Fluorescence In Situ Hybridization) was carried out to identify the origin of the extra chromosomal material attached to 5p15. The extra chromosomal material was found to be derived from chromosome 13. The cytogenetic study of her father identified a translocation (5;13)(p15;q22) and the propositus' karyotype was redesigned as 46,XX,der(5)t(5;13)(p15;q22)pat (fig. 4A and 4B).

## Discussion

The majority of trisomy 13q cases (>91%) are maternal in origin and, similar to other autosomal trisomies, the extra chromosome is typically due to errors in meiosis I. Surprisingly, however, a large number of errors also occur during maternal meiosis II (approximately 37%), distinguishing trisomy 13 from other acrocentric and most nonacrocentric chromosomes. As other trisomies, failure to recombine is an important contributor to nondisjunction of chromosome 13 [9]. Many cases arise from parental balanced translocations, sometimes from parental pericentric inversions and rarely from de novo duplications [10-12]. The presented case suffered a chromosomal unbalance with a partial trisomic component 13q22-qter and a monosomic component 5p15-pter from paternal origin. The length defect in chromosome 5 was short and the patient did not exhibit phenotypic features associated to deletions in 5pter, like *cri du chat *syndrome or speech disabilities, hence the material responsible for the phenotype changes is supposed to be the material extra in chromosome 13 [13]. It is known that some chromosomal diseases show variability of phenotype. This patient, as some others previously reported cases with partial trisomy 13, had hypoacusia, hypochromic anaemia [14] or splenomegaly [15], but she did not have polydactyl [16] or characteristic eyelashes or ears [17]. By contrast, she suffered leukoencephalopathy, minimal changes in sterols, early menopause and late onset generalized seizures.

The reduction of available cholesterol and total cholesterol in lymphocytes (both in the membrane and intracellular) provokes indirectly a reduction in the long chain of fatty acids. This may contribute to decrease of estradiol and subsequent early menopause.

Seizures in unbalanced trisomy of chromosome 13 have been described to occur at early age, but usually associated to structural lesions. In other chomosomopathies seizures are due to dysfunctional neuronal GABA transporters or another neurotransmition regulation [18,19]. In previous reports no details are given on the type of seizure or EEG findings. In genetic leukodystrophy, clinical seizures are mainly of focal origin. At the beginning, the EEG is normal or shows mild slowing with progressive slowing and focal or multifocal paroxysmal discharges in later stages [20]. By contrast, our patient showed atonic o myoclonic generalised seizures with normal background activity and generalised paroxysmal discharges on EEG. She had good response to valproic acid leading to normalization on follow-up EEGs.

The normal motor conduction speed and the normal long chain fatty acid in fibroblasts discard adrenoleukodystrophy. To date, the only gene described for leukoencephalopathy with vanishing white matter (VWM) codes for oligodendrocyte-specific protein (OSP) and is located on chromosome 3q27 [21]. We do not know if the leukoencephalopathy changes in the MRI could be related to the lipidic deficit. Knoblauch H. et al. [22] described a cholesterol lowering gene localised in chromosome 13q. However, we think that our patient had other specific characteristics; she did not suffer familiar hypercholesterolemia or increase of LDL cholesterol.

Today, the diagnosis of leukoencephalopathies is more frequent because the use of neuroimaging represents an early diagnostic tool in clinical evaluation. Many cases have identifiable clinical, biochemical, molecular or neuroimaging markers. However identification and categorisation is still limited in a considerable number of cases by the lack of biological or genetic markers. Molecular techniques will facilitate the delineation of phenotypic variability and the understanding of the biological basis for future rational therapeutic interventions.

## Conclusion

The presented case illustrates the phenotype of 13q22 in adult age at the time that reaffirm the importance of studying the karyotype of any patient with seizures who shows special clinical features including leukoencephalopathy, stroke at a young age, fatty acids disturbances, microcephaly, hypotelorism, short neck, hemangiomata, short fingers or distal swell in thumbs.

## List of abbreviations

LDL: Low Density Lipoprotein; EEG: Electroencephalogram; MRI: Magnetic Resonance Imaging; FISH: Fluorescence In Situ Hybridization; NREM: No Rapid Eye Movement; VWM: Vanishing White Matter; OSP: Oligodendrocyte-Specific Protein.

## Competing interests

The authors declare that they have no competing interests.

## Authors' contributions

RR described the phenotypic features and complementary tests findings of the case. MMG and JS contributed to the manuscript writing and discussion. I. Hernando performed the cytogenic and genomic analyses. M Giros performed the biochemical studies on lipids.

## Consent

Written informed consent was obtained from the patient for publication of the case report and accompanying images.
